# Gut Metabolite Urolithin A Inhibits Osteoclastogenesis and Senile Osteoporosis by Enhancing the Autophagy Capacity of Bone Marrow Macrophages

**DOI:** 10.3389/fphar.2022.875611

**Published:** 2022-05-12

**Authors:** Huaqiang Tao, Yunxia Tao, Chen Yang, Wenming Li, Wei Zhang, Xueyan Li, Ye Gu, Yujing Hong, Huilin Yang, Yu Liu, Xing Yang, Dechun Geng

**Affiliations:** ^1^ Department of Orthopedics, The First Affiliated Hospital of Soochow University, Suzhou, China; ^2^ Anesthesiology Department, Suzhou Municipal Hospital (North District), Nanjing Medical University Affiliated Suzhou Hospital, Suzhou, China; ^3^ Department of Orthopedics, Changshu Hospital Affiliated to Soochow University, First People’s Hospital of Changshu City, Changshu, China; ^4^ Department of Preventive Medicine, Nantong University, Nantong, China; ^5^ Department of Orthopedics, Wuxi Ninth People’s Hospital Affiliated to Soochow University, Wuxi, China; ^6^ Orthopedics and Sports Medicine Center, Suzhou Municipal Hospital (North District), Nanjing Medical University Affiliated Suzhou Hospital, Suzhou, China

**Keywords:** Urolithin A, senile osteoporosis, osteoclast, autophagy, MAPK

## Abstract

Senile osteoporosis (SOP) is a systemic bone disease that is significantly associated with age and eventually leads to deteriorated bone strength and increased fracture risk. Urolithin A (Uro-A) is a gut microbiome-derived compound that is mainly produced from pomegranates and some nuts. Uro-A has attracted great attention in recent years in view of its protective effects on aging-related diseases, including muscle dysfunction, kidney disease and knee injury. However, its protective influence and possible mechanisms in senile osteoporosis remain unclear. Our study describes the beneficial effect of Uro-A on bone marrow macrophages (BMMs). The *in vitro* results demonstrated that Uro-A inhibited receptor activator for nuclear factor-κB ligand (RANKL)-induced osteoclastogenesis in BMMs in a concentration-dependent manner. Uro-A significantly reduced the expression of osteoclast-related genes and bone resorption. Mechanistically, we found that the autophagy ability of BMMs was significantly enhanced in the early stage of Uro-A treatment, accompanied by the activation of LC3 and Beclin 1. At the same time, this enhanced autophagy activity was maintained until the later stage after stimulation with RANKL. Furthermore, we found that the MARK signaling pathway was blocked by Uro-A treatment. In a mouse model of aging, Uro-A effectively inhibited bone loss in the proximal femur, spine and tibia of aging mice. These results indicated that Uro-A is a robust and effective treatment for preventing senile osteoporosis bone loss.

## Graphical Abstract

Uro-A, which is a metabolite of intestinal flora and mainly produced from pomegranates and some nuts, suppresses osteoclast formation and bone resorption by activating autophagy. Uro-A upregulated the expression of Beclin 1 and LC3 and modulated bone homeostasis 4 in BMMs, thus resulting in decreased osteoclast-related markers and bone loss.
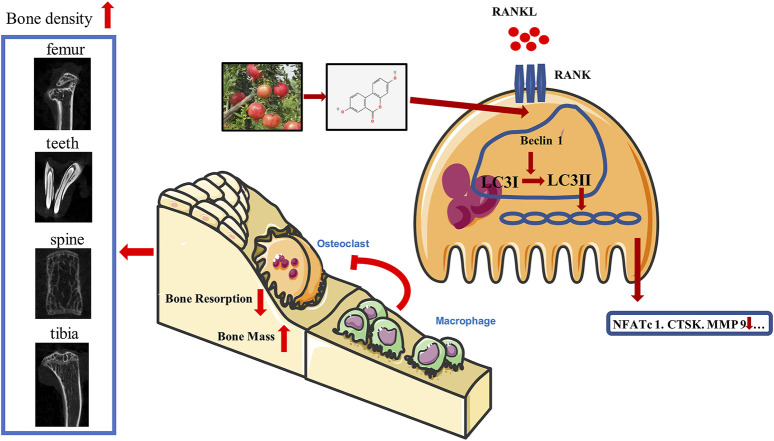

## Introduction

Osteoporosis is a systemic metabolic bone disease, and its etiology has been determined over the course of many years (Macías et al., 2021). Senile osteoporosis (SOP) often occurs in people over 70 years old and is an inevitable degenerative disease with aging. SOP is a disorder of bone metabolism that features disturbed bone microstructures, reduced trabeculae and thinning of cortical bone, resulting in a reduction in bone strength and an increase in susceptibility to fracture. According to statistics, the population of people over 60 will account for approximately 22% of the total population worldwide. With the extension of life expectancy and the continuous increase in the elderly population, SOP, one of the difficult issues of middle-aged and old people, has become a serious social problem (Pardanani, 2021; Wan et al., 2021).

Bone loss in SOP patients generally involves the entire skeletal system, and the degree of bone loss varies in different parts of the body. With age, bone resorption gradually exceeds bone formation, and eventually, bone mass is gradually reduced, leading to SOP (Rodríguez et al., 2021). Osteoclasts are composed of multinucleated giant cells, which have a special absorption function and are engaged in the process of bone resorption (Li, 2021). The main function of osteoclasts is to absorb mineralized bone, dentin and calcified cartilage (Kobari et al., 2021; de Melo Pereira et al., 2022; Jin et al., 2022). It is currently believed that osteoclasts are the only cells that can absorb bone. However, once certain external conditions are out of balance, a surge of osteoclasts accelerates the erosion of bone and eventually leads to irreversible bone loss (Kornel et al., 2021). Therefore, inhibiting the excessive activation of osteoclasts is an effective strategy for the treatment of osteoporosis.

Uro-A is a natural gut microbiome-derived metabolite that is produced from ingested ellagitannins (ETs) and ellagic acid (EA). There are four types of urolithins (Uro-A, Uro-B, Uro-C, Uro-D), and Uro-A is the most common species (D’Amico et al., 2021; Wang et al., 2021). It can be synthesized by intestinal microbes in the colon by converting polyphenols. However, only 40% of elderly individuals can convert dietary fiber into Uro-A (Singh et al., 2021). This conversion depends on the attributes of the individual’s intestinal flora, biological health conditions and eating habits. Uro-A has been proven to have powerful anti-inflammatory and antioxidant effects (Żary-Sikorska et al., 2020; Abdelazeem et al., 2021; Hasheminezhad et al., 2021; Yi et al., 2021). Previous studies demonstrated that Uro-A reduced colon inflammation and improved intestinal barrier integrity in DSS-induced mouse models of colitis by reducing the cytokines interleukin 1 beta (IL-1β), interleukin 6 (IL-6), and tumor necrosis factor alpha (TNFα). Similarly, these inflammatory factors are reduced in a Uro-A-treated streptozotocin-induced diabetic mouse model (Singh et al., 2019). In recent years, Uro-A has been shown to effectively improve the decline of body functions caused by aging and has attracted much attention (Andreux et al., 2019; Djedjibegovic et al., 2020). Ryu et al. (2016) found that mice exhibited stronger muscle strength after Uro-A supplementation. In Xia’s research, Uro-A administration was proven to increase running activity in C57BL/6J mice (Xia et al., 2020). However, its protective influence on senile osteoporosis bone loss needs to be fully explored.

The effect of Uro-A on osteoclast differentiation has been reported previously (Tao et al., 2021; Wang et al., 2022), and Uro-A was revealed to alleviate RAW264.7-induced osteoclasts by downregulating the inflammatory cascade. In this study, we explored the effect of Uro-A on bone marrow macrophages (BMMs) in mice. After RANKL and M-CSF induced BMMs to differentiate into osteoclasts, we found that Uro-A inhibited the production of osteoclasts in a concentration-dependent manner. Considering the powerful autophagy-promoting effect of Uro-A in other tissues and cells, we explored its effect on autophagy-related functions in BMMs. Through transmission electron microscopy analysis, we found that after adding Uro-A, more goblet-shaped autophagosomes appeared in the cytoplasm of BMMs, which indicated that Uro-A promoted cell autophagy. After stimulation with RANKL, Uro-A further promoted the enhanced autophagy effect. In aged mice, we found that oral administration of Uro-A effectively alleviated bone loss of the mouse proximal femur, spine and tibia. This shows that Uro-A may be a promising treatment to prevent senile osteoporosis.

## Methods and Materials

### Drugs and Reagents

Uro-A dissolved in dimethyl sulfoxide was purchased from Tokyo Kasei Kogyo (Tokyo, Japan) and subsequently diluted to 100 μM. Recombinant RANKL and macrophage colony stimulating factor (M-CSF) were obtained from R&D Systems (Minneapolis, United States). AS1842856 was purchased from MedChem Express (Princeton, United States). Alpha-modified minimal essential medium (a-MEM, for culturing bone marrow-derived macrophages) and fetal bovine serum (FBS) were purchased from Thermo Fisher Scientific (St. Louis, MO, United States).

### Cell Culture

Fresh murine bone marrow-derived macrophages (BMMs) were used to induce osteoclast formation in our experiment. BMMs were isolated from the murine femur in C57BL/6 mice (8 weeks) as described previously (Zhou et al., 2017). Nonadherent cells were then collected in the suspension and cultured in a suitable mixed medium (α-MEM/10% FBS/50 ng/ml M-CSF) for 3 days to obtain pure BMMs. After an additional 3 days in culture, the cells were collected and used for experimental purposes. A humidified incubator at 5% CO_2_ was used to maintain BMM growth.

### Cytotoxicity Assay

BMMs (6 × 10^3^) were reseeded into 96-well plates and incubated with the mixed medium overnight to adhere. After half of the day, different concentrations of Uro-A were added to each well and incubated for 24 and 48 h. MTS solution (ApexBio, Boston, United States) was added to the plates and continue to incubate for 2 h. The cytotoxicity of Uro-A was evaluated by measuring the optical density (OD) at 450 nm with a microplate reader (BioTek, VT, United States).

### *In Vitro* Osteoclast Formation Assay

BMMs flushed from femurs were grown in the mixed medium in 24-well plates at a density of 9 × 10^4^ to induce osteoclastogenesis. Cells were treated with different concentrations of Uro-A (5 and 10 μM) or left untreated, after which they were cultured in medium containing 50 ng/ml RANKL and M-CSF for 4–5 days until osteoclastic differentiation occurred. The medium was replaced every 2 days. Ultimately, the cells were fixed with paraformaldehyde (4 wt%, Biosharp, Guangzhou, China) for 15 min after rinsing with phosphate-buffered saline, and a leukocyte acid phosphatase staining kit (Sigma–Aldrich) was used to stain the osteoclasts. TRAcP+ cells (≥3 nuclei) with more nuclei and large volumes were classified as osteoclasts and counted using a fluorescence microscope (Axio Imager 2, Zeiss).

### F-Actin Staining

After inducing osteoclasts as described above, the cells were fixed with a 4% formaldehyde solution for 15 min at 4°C, and 0.1% Triton X-100 (Beyotime, Shanghai, China) was added to permeabilize the cells. Then, the cells were blocked with QuickBlock Blocking Solution (Beyotime) for 1 h. The processed samples were incubated with phalloidin for 1 h at 37°C. Finally, the cell nuclei were stained with DAPI (Beyotime) for 10 min, and images of the F-actin ring and nuclei were then acquired with a fluorescence microscope (Zeiss, Dresden, Germany) at a 320× magnification. The number of nuclei were assessed using ImageJ.

### Resorption Pit Assay

A resorption pit assay was employed to test the osteoclastic absorption function. BMMs were plated at a density of 1 × 10^4^ cells/well in 48-well plants with Osteo Assay Plates (Yaoang Biological Technology Co., Ltd., Shanghai, China). Subsequently, samples were treated with Uro-A (0, 5, and 10 μM) and 50 ng/ml RANKL and M-CSF to generate mature osteoclasts as mentioned above. After 5 days, a scanning electron microscope (Leica, Germany) was employed to observe the morphology of osteoclasts and absorption area during differentiation.

### Western Blot Analysis

After treatment with Uro-A, RIPA buffer (Beyotime) was used to lyse BMMs for harvesting total protein. We used SDS-polyacrylamide gels (Beyotime) to separate the proteins in the samples. Subsequently, proteins were transferred to PVDF membranes (Merck Millipore, MA, United States) and blocked with QuickBlock™ blocking buffer (Beyotime) for 1 h at room temperature. After incubation with primary antibodies overnight, PVDF membranes were rinsed three times with TBST (NCM Biotech, Suzhou). Then, the corresponding secondary antibodies (Beyotime) were added for another 1 h. We used an ECL reagent (Epizyme, Sigma–Aldrich) to detect antibody-antigen complexes. The following primary antibodies were used in our experiment: cathepsin K (CTSK; ab19027), nuclear factor of activated T cells 1 (NFATc1; ab25916), MMP9 (ab228402), ULK1 (ab203207), Beclin 1 (ab210498), LC3 (ab192890) and β-actin (ab6276), which were purchased from Abcam (Cambridge, United Kingdom). In addition, primary antibodies against ERK (#4695), phospho-ERK (#4370), JNK (#9252), phospho-JNK (#9255), P38 (#8690), and phospho-P38 (#4511) were provided by Cell Signaling Technology (Boston, United States).

### Quantitative RT-PCR

We used TRIzol reagent (Beyotime) to harvest the RNA from BMMs under different intervention conditions. Then, the samples were extracted with chloroform. Processed RNA was centrifuged at a speed of 12,000 for 15 min. The upper layer of transparent liquid was collected, and we added an equal volume of isopropanol for the follow-up experiment. RNA from BMMs was reverse transcribed into cDNA by using PrimeScript RT Master Mix (Takara, Dalian, Japan), and the gene expression levels of CTSK, MMP9, NFATc1, OSCAR, Atp6v0d2, Beclin 1 and ULK1 were measured by qRT–PCR (S1000, Bio-Rad, United States) using a mixture of dNTP reagent (TaKaRa, Japan), the forward and reverse primers and RNase-free H_2_O (Abcam). The forward and reverse primers are listed in Table 1. The β-actin gene was used as the housekeeping gene. Finally, 2^−ΔΔCt^ method was employed to quantify the relative expression levels of mRNA.

**TABLE 1:** Sequences of mouse primers used in qRT-PCR.

Genes	Forward (5′–3′)	Reverse (5′–3′)
NFATc1	CAA​CGC​CCT​GAC​CAC​CGA​TAG	GGC​TGC​CTT​CCG​TCT​CAT​AGT
CTSK	GGGAGAAAAACCTGAAGC	ATTCTGGGGACTCAGAGC
Acp 5	TGTGGCCATCTTTATGCT	GTCATTTCTTTGGGGCTT
OSCAR	CGAAGGTTCTGGCTCCT	CCTGCTGTGCCAATCAC
Atp6v0d2	TGG​CCT​CAT​ACG​TTC​ATT​T	TTTGAGCTTGGGGAGAAG
ULK1	CCGAGGGCTGTGTACC	TGGTCCGTGAGAGTGTGC
Beclin 1	CTG​TAG​CCA​GCC​TCT​GAA​A	CCTCTTCCTCCTGGGTCT
β-actin	CGT​TGA​CAT​CCG​TAA​AGA​CC	AAC​AGT​CCG​CCT​AGA​AGC​AC

### Cellular Immunofluorescence Staining

For immunofluorescence staining, BMMs obtained from fresh marrow were plated in 24-well plates for the indicated times with or without Uro-A treatment. Then, the cells were rinsed with PBS 3 times and fixed with 4% paraformaldehyde for 15 min. Triton X-100 was used to permeabilize cells for 10 min. Furthermore, BMMs were stained with primary antibodies against LC3 (ab192890, Abcam) and Beclin 1 (ab62557, Abcam) at 4°C for 12 h, and the corresponding secondary antibody and goat anti-rabbit IgG H&L (ab150077, abcam) were incubated for 1 h in the dark. After BMMs were stained with DAPI for 10 min, we collected the samples and used a confocal microscope (Zeiss, Dresden, Germany) to observe the cells.

### Transmission Electron Microscopy

The autophagosomes of the BMMs were observed by transmission electron microscopy (TEM) (HT7700, Japan). In brief, BMMs treated with or without Uro-A for 4 h were collected, centrifuged, washed, and fixed with 2.5% glutaraldehyde. We used an ethanol gradient for dehydrating samples. Then, the processed cells were embedded in resin and sectioned. TEM was used to observe intracellular structure after the ultrathin sections were stained with uranyl acetate.

### Mouse Model of Senior OP and Uro-A Administration

The animal experiments used in this study were conducted under the regulations of the Ethics Committee of the First Affiliated Hospital of Soochow University. Twenty-eight female C57BL/6 mice aged 10 weeks were purchased from the Animal Center of Soochow University and housed in a standard animal room. The experimental mice were randomly divided into four groups (seven mice per group). The mice fed for 20 weeks were treated as the sham group. The rest of the mice were routinely fed to 70 weeks. Uro-A was dissolved in 0.5% sodium carboxymethyl cellulose (0.5% CMC-Na; Sigma–Aldrich). The mice in the vehicle group were received an equal volume of 0.5% CMC-Na solution after 50 weeks. Mice in the low Uro-A administration group were received 10 mg/kg Uro-A, and mice in the high Uro-A administration group were received 20 mg/kg Uro-A. For the Uro-A-intervention group, mice received intragastric administration four times a week for 20 consecutive weeks. When fed to 70 weeks, the mice were euthanized and received further processing.

### Micro-CT (μCT) Analysis

When reaching a certain growth cycle, each group of mice was euthanized with a small animal anesthesia machine (RWD Life Science, Shenzhen, China). Proximal femur, tibias, teeth and spinal cord segments were removed from the body and collected in a formalin solution. A high-resolution micro-CT system (SkyScan1176, Aartselaar, Belgium) was used to scan the microstructure of the bone tissue with the scanning parameters set to a high resolution (9 μm). Three-dimensional (3D) structural images of the proximal femur and 2D images of teeth, tibias and spinal cord segments were reconstructed to evaluate body bone mass in each group of mice. Bone mineral density (BMD, g/cm^3^), bone volume ratio (BV/TV, %), trabecular number (Tb.N, 1/mm), bone volume (BV, mm^3^), trabecular separation (Tb.Sp, mm) and trabecular thickness (Tb.Th, mm) were systematically evaluated.

### Bone Histomorphometry Analysis

After scanning the CT, proximal femurs were collected and decalcified in 10% ethylenediaminetetraacetic acid (Sigma–Aldrich, Australia) at room temperature for 21 days, after which paraffin was used for embedding. Next, histological slices with a thickness of 5 μm were prepared. The samples were dewaxed with xylene and subjected to gradient hydration. Hematoxylin and eosin (H&E) staining and TRAcP activity staining were performed as described previously (Li et al., 2021). Images were obtained using an Axiovert 40C optical microscope (Zeiss, Germany).

### Statistical Analysis

All data in this experiment were analyzed by using SPSS 25.0 statistical software, using the mean ± standard deviation, a statistical method using one-way analysis of variance (ANOVA), and pairwise comparisons between groups using the LSD-t test. A difference at *p < 0.05 was considered significant, and a difference at **p < 0.01 was considered highly significant.

## Results

### Uro-A Inhibited RANKL-Induced Osteoclast Formation in BMMs

**FIGURE 1:**
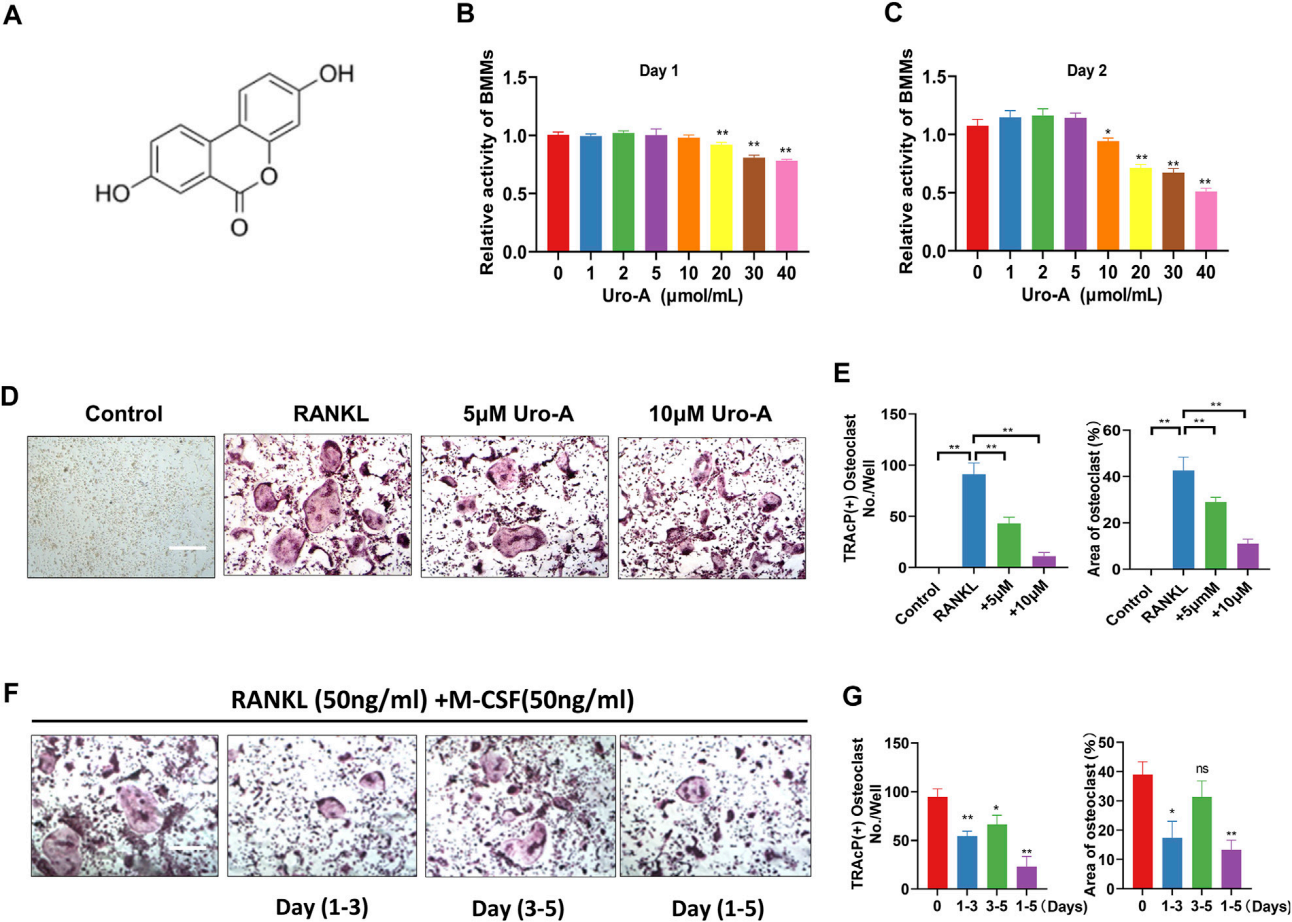
Uro-A inhibited RANKL-induced osteoclast formation in BMMs. **(A)** The chemical structure of Uro-A. **(B,C)** CCK-8 analysis was performed with various concentrations of Uro-A in BMMs for 24 and 48 h. **(D)** Representative images of TRAcP staining showing that Uro-A inhibited osteoclastogenesis dose-dependently with BMMs. **(E)** Quantification of the TRAcP-positive multinucleated cells (nuclei > 3) and area of osteoclast (%). **(F)** Representative images of TRAcP staining showing BMMs treated with Uro-A for the indicated days during osteoclastogenesis. **(G)** Quantification of TRAcP-positive multinucleated cells treated with Uro-A in different time periods and area of osteoclast (%). All bar graphs are presented as the mean ± SD. *p < 0.05, **p < 0.01. Scale bar = 200 μm; *n* = 3 per group. RANKL, receptor activator of nuclear factor-κB ligand; TRAcP, tartrate-resistant acid phosphatase.

Figure 1A shows the chemical structure of Uro-A. To determine the cytotoxic effects of Uro-A on BMMs, a CCK-8 kit was used to detect its influence on cell viability after 24 and 48 h, and the results indicated that Uro-A had little effect on cell viability at concentrations lower than 10 μM compared to the untreated group (Figures 1B,C). To investigate the effect of Uro-A on osteoclastogenesis, we chose concentrations of 5 and 10 μM to intervene in the differentiation of BMMs. Strikingly, the number of osteoclasts surged after the addition of RANKL, and Uro-A inhibited osteoclast formation in a concentration-dependent manner (Figures 1D,E). To explore which stage of osteoclast formation is influenced by Uro-A, BMMs treated with 50 ng/ml RANKL and 50 ng/ml M-CSF were treated with 10 μM Uro-A for the indicated times. TPAcP staining revealed that after treatment with RANKL, Uro-A significantly suppressed osteoclast formation in BMMs at the early stage (DAY 1–3) but not at the mid-late stage (DAY 3–5) (Figures 1F,G).

### Uro-A Inhibited Bone Resorption and Osteoclast-Related Markers in BMMs

**FIGURE 2:**
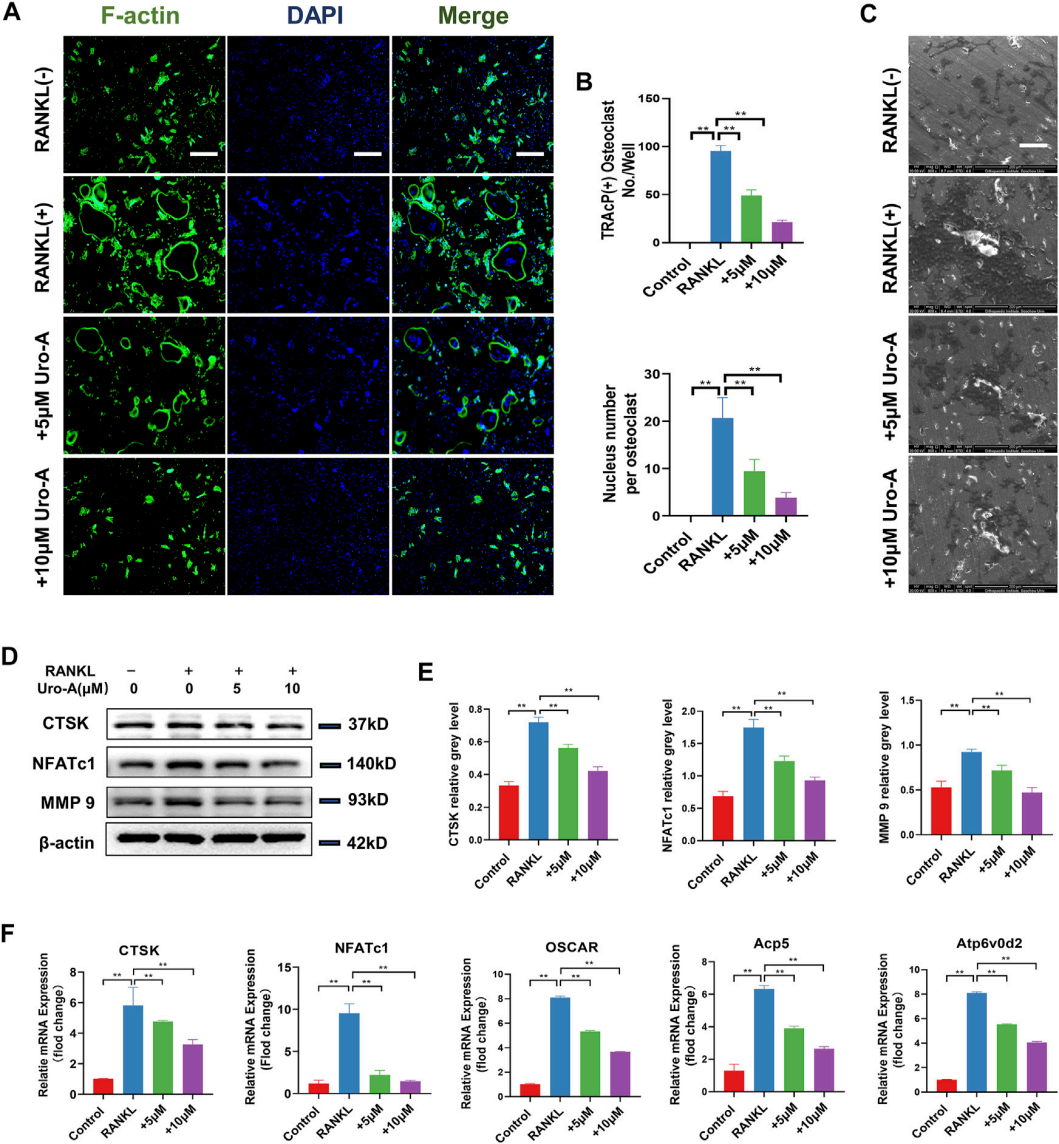
Uro-A inhibited F-acting ring formation and osteoclast-related markers in BMMs. **(A)** F-actin (green) and nuclei (blue) staining of osteoclasts were photographed by fluorescence microscope. **(B)** Quantification of the nuclear aggregation number per osteoclast and relative number of osteoclasts. **(C)** BMMs were plated on bone resorption plates and cultured with induction medium containing 50 ng/ml RANKL and M-CSF together with various concentrations of Uro-A (0, 5 and 10 μM) for 4 days. **(D,E)** Representative Western blot images of the effects of Uro-A on CTSK, NFATc1 and MMP9 expression. **(F)** BMMs were incubated with induction medium containing 50 ng/ml RANKL, M-CSF and various concentration of Uro-A for 24 h. The gene copies of Acp5, CTSK, OSCAR, NFATc1 and Atp6vo2d were quantified by RT-PCR. All bar graphs are presented as the mean ± SD. *p < 0.05, **p < 0.01. Scale bar = 200 μm; *n* = 3 per group. RANKL, receptor activator of nuclear factor-κB ligand; TRAcP, tartrate-resistant acid phosphatase.

The F-actin ring clearly shows the structural distribution of microfilaments in osteoclasts, and it is considered to be highly related to the function of osteoclasts (Xiao et al., 2021). In our research, BMMs treated with or without Uro-A were costained with F-actin and DAPI to detect osteoclast size and nuclear number. As shown, after treatment with 50 ng/ml RANKL and M-CSF, the number of osteoclasts with clear outlines increased intensely. Uro-A significantly reduced the size of F-actin rings and nuclear aggregation in BMMs relative to the RANKL group (Figures 2A,B). Because mature osteoclasts exhibit strong bone absorptive capability, we used a hydroxyapatite-coated plate to check whether Uro-A inhibits bone resorption. As shown in Figure 2C, treatment with 50 ng/ml RANKL and M-CSF promoted resorbing activity, but an increasing concentration of Uro-A obviously reduced the resorption area. Then, we investigated whether Uro-A blocks the expression of osteoclast-related markers during the differentiation of BMMs. The western blot results showed that CTSK, NFATc1 and MMP9 expression was suppressed after treatment with Uro-A compared with the RANKL group (Figures 2D,E). Similarly, the qRT–PCR results showed that the expression of osteoclast differentiation genes, including CTSK, NFATc1, OSCAR, Acp5 and Atp6v02d, was downregulated after Uro-A treatment, and an increasing concentration of Uro-A effectively blocked the expression of these genes, which was consistent with the western blot data (Figure 2F). Convincingly, Uro-A is an effective osteoclast inhibitor agent in RANKL-stimulated cells.

### Uro-A Promoted the Autophagy Ability of BMMs *In Vitro*

**FIGURE 3:**
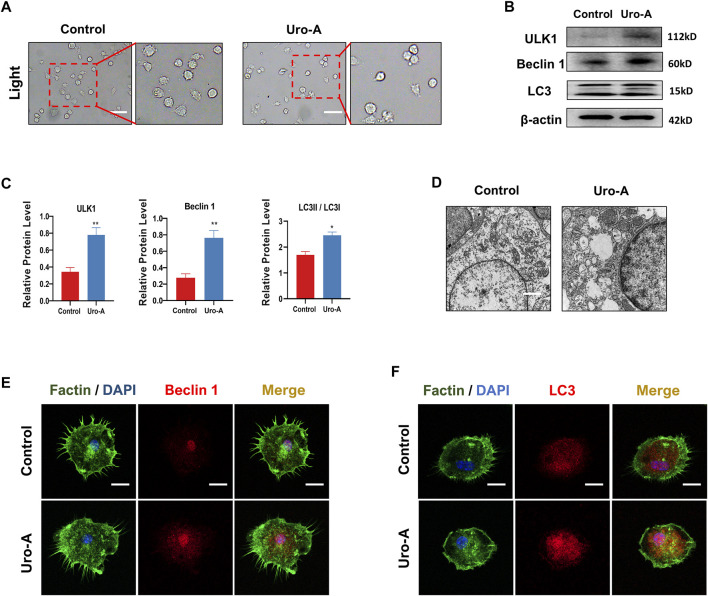
Uro-A promoted the autophagy ability of BMMs *in vitro*. **(A)** After presentative treatment with 10 μM Uro-A for 4 h, BMMs were visualized at the magnification of 320× under an inverted microscope. **(B,C)** Representative Western blot images and quantitative analysis of the effects of Uro-A on ULK1, Beclin 1 and LC3 expression. **(D)** The structure of BMMs under transmission electron microscope. Scale bar = 2 μm. **(E,F)** After treated with or without 10 μM Uro-A, BMMs were stained for Beclin 1 (red) and LC3 (red) antibody and secondary antibody with FITC (green). And the localization was visualized using a confocal microscope. All bar graphs are presented as the mean ± SD. *p < 0.05, **p < 0.01. Scale bar = 200 μm; *n* = 3 per group.

Previous research indicated that cell autophagy may be related to bone homeostasis (Bhutia, 2021). Uro-A has been proven to modulate autophagy in both cultured cells and mouse brains. In our study, we further explored the influence of Uro-A on BMMs autophagy. We used 10 μM Uro-A to treat BMMs for 4 h. Figure 3A reveals that the BMMs appeared round and had a dispersed morphology on the first day of cell culture. When treated with Uro-A, the shape and viability of the BMMs were not affected. Autophagy is modulated by a family of highly conserved genes known as autophagy-related genes, which has approximately 20 members. Among these genes, Beclin 1 is usually known as a crucial autophagy marker for the formation of phagophores. Usually, LC3 is linked to lipids or phosphatidylethanolamine to form LC3II, which integrates into the phagophore membrane and plays an important role during autophagy (Ajazi et al., 2021; da Silva Lima et al., 2021). Western blot analysis showed that Uro-A enhanced the expression of ULK1, LC3 and Beclin 1 compared with the control group (Figures 3B,C). Through TEM, we observed the presence of a small amount of scattered autophagic vacuoles in the control group and double-membraned autophagic vesicles containing needle-like structures resembling crystals in the Uro-A-treated group (Figure 3D), suggesting that Uro-A amplifies the autophagy effect in BMMs. In addition, the fluorescence intensity of Beclin 1 and LC3 apparently increased in the Uro-A group (Figures 3E,F). This further indicates that Uro-A affects the autophagy in BMMs.

### Uro-A Enhanced Autophagy in RANKL-Induced Osteoclastogenesis *In Vitro*

**FIGURE 4:**
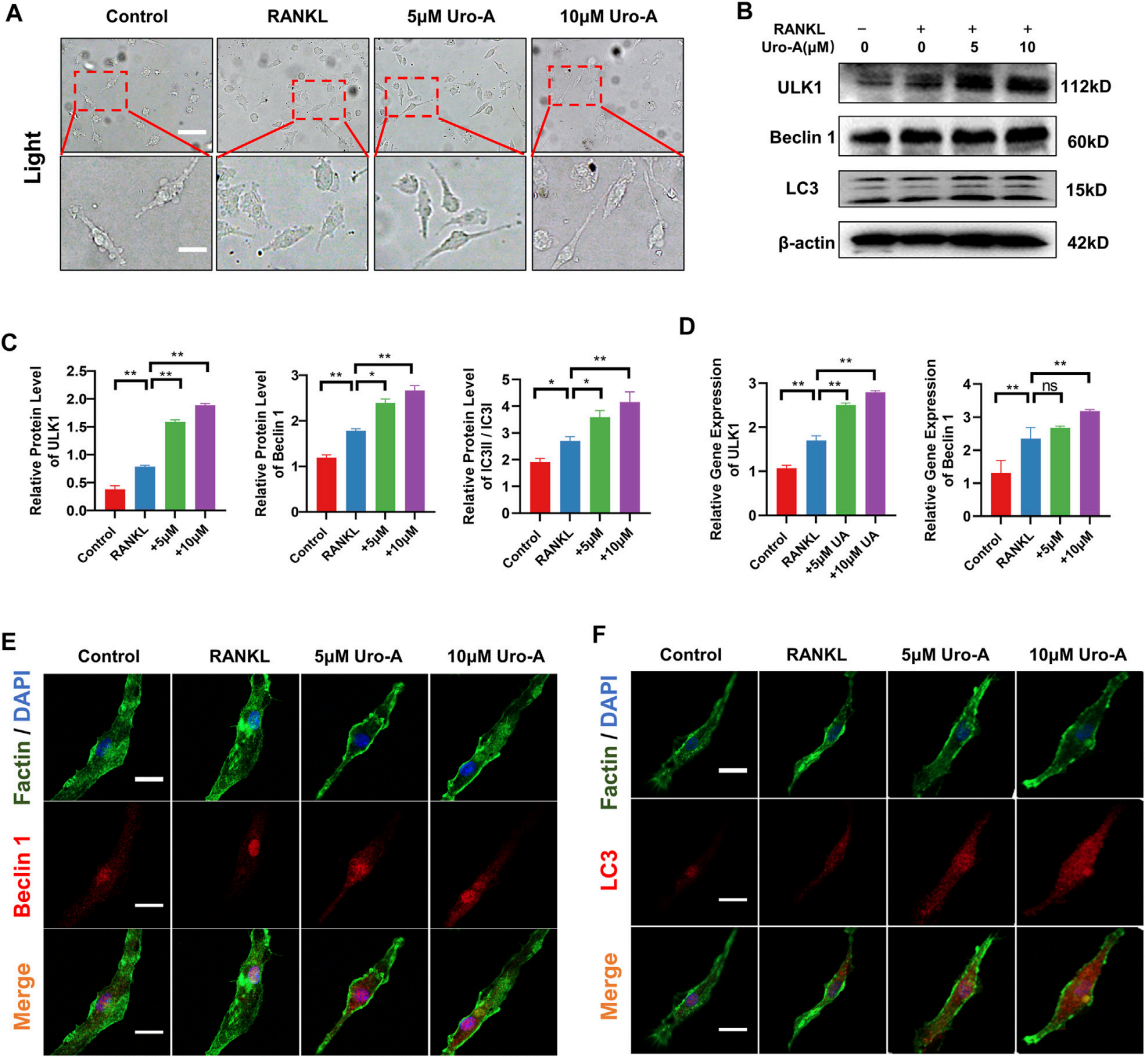
Uro-A also enhanced autophagy in RANKL-induced osteoclastogenesis *in vitro*. **(A)** After presentative treatment with 50 ng/ml RANKL, M-CSF and 10 μM Uro-A for 3 days, BMMs were visualized at the magnification of 320× under an inverted microscope. **(B,C)** Representative Western blot images and quantitative analysis of the effects of Uro-A on ULK1, Beclin 1 and LC3. **(D)** The gene copies of ULK1 and Beclin 1 in each group were quantified by RT-PCR. **(E,F)** After treated with or without 10 μM Uro-A for 3 days, BMMs were stained for Beclin 1 (red) and LC3 (red) antibody and secondary antibody with FITC (green). And the localization was visualized using a confocal microscope. All bar graphs are presented as the mean ± SD. *p < 0.05, **p < 0.01. Scale bar = 200 μm; *n* = 3 per group.

To further explore the impact of Uro-A on BMMs after treatment with RANKL and M-CSF, we conducted a series of experiments. First, after RANKL treatment for 3 days, we observed that BMMs had a clustered morphology. Compared with the culture for 1 day, most of the cells showed a fusiform elongated shape (Figure 4A). This may be caused by environmental factors in the culture or an evolution of the cell itself. With the addition of Uro-A, we did not find too many morphological differences compared with the RANKL group. Next, western blot analysis showed that RANKL dramatically increased the expression of ULK1, Beclin 1 and LC3. The expression of Beclin 1 and LC3 was further enhanced after Uro-A administration (Figures 4B,C), which was consistent with the RT–PCR results (Figure 4D). We used 10 μM Uro-A to treat BMMs for 3 days. Cellular immunofluorescence results showed that the expression of Beclin 1 and LC3 was upregulated after exposure to RANKL, and an increasing concentration of Uro-A further enhanced this effect. Thus, Uro-A inhibited RANKL-triggered osteoclast formation, which was accompanied by autophagy-related indicators.

### Uro-A Inhibited Osteoclast Formation by Suppressing MAPK Signaling Pathway

**FIGURE 5:**
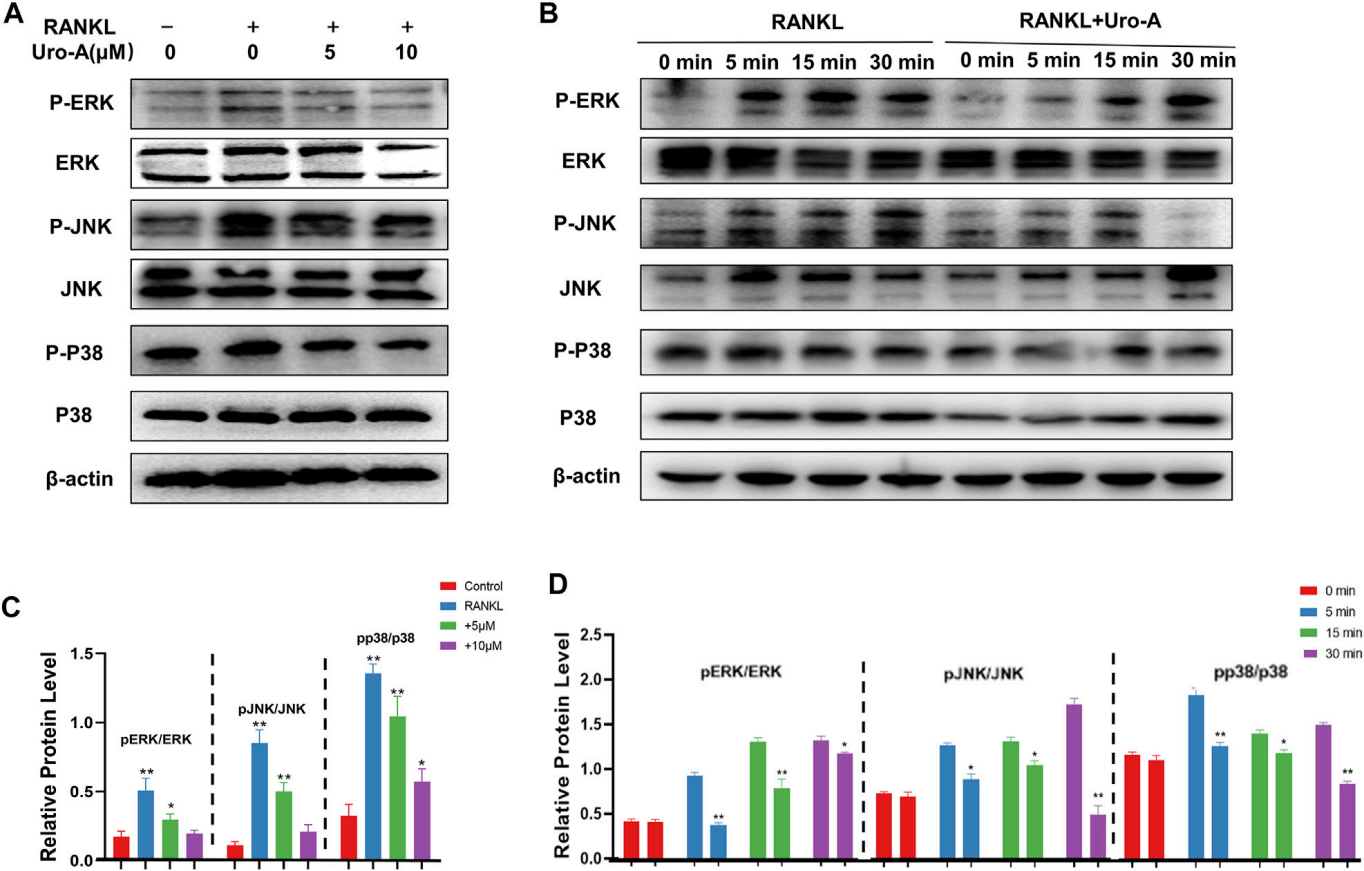
Uro-A inhibited osteoclast formation *via* MAPK signaling pathway. **(A,C)** Western blot images of JNK, ERK, P38 and phosphorylation of JNK, ERK, P38 stimulated by 50 ng/ml RANKL and M-CSF with or without Uro-A treatment for 12 h. Grayscale value ratio of p-JNK/JNK, p-ERK/ERK and p-P38/P38. **(B,D)** BMMs were treated for 12 h with Uro-A, after which they were treated for 0–30 min with RANKL (50 ng/ml) and M-CSF (50 ng/ml). Western blotting was then used to measure ERK, JNK and p38 expression and phosphorylation. Western blotting data were quantified respectively. *p < 0.05, **p < 0.01. *n* = 3 per group.

The MAPK signaling pathway, which contains ERKs, JNKs and P38, is known to play a critical role in cell function and has been verified as a driving factor for osteoclast maturity (He et al., 2022). Additionally, many scientists have previously confirmed that the MAPK cascade is related to the occurrence of osteoclastic autophagy (Xu et al., 2020). To further investigate the mechanism by which Uro-A suppresses osteoclastogenesis, we evaluated the influence of Uro-A on the MAPK signaling pathway. Western blot analysis showed that p-ERK, p-JNK and p-P38 were highly expressed after RANKL and M-CSF stimulation, leading to higher ratios of p-ERK/ERK, p-JNK/JNK and p-P38/P38. However, Uro-A inhibited the phosphorylation of the MARK family (Figures 5A,C). In the first 30 min of RANKL treatment, the expression of p-ERK, p-JNK and p-P38 gradually increased, Uro-A pretreatment started to significantly inhibit p-ERK signaling and p-p38 signaling in 5 min and p-JNK signaling in 30 min (Figures 5B,D). These results suggest an inhibitory influence of Uro-A on the MAPK signaling pathway.

### Uro-A Inhibited Systematic Bone Loss in Aging Mice

**FIGURE 6:**
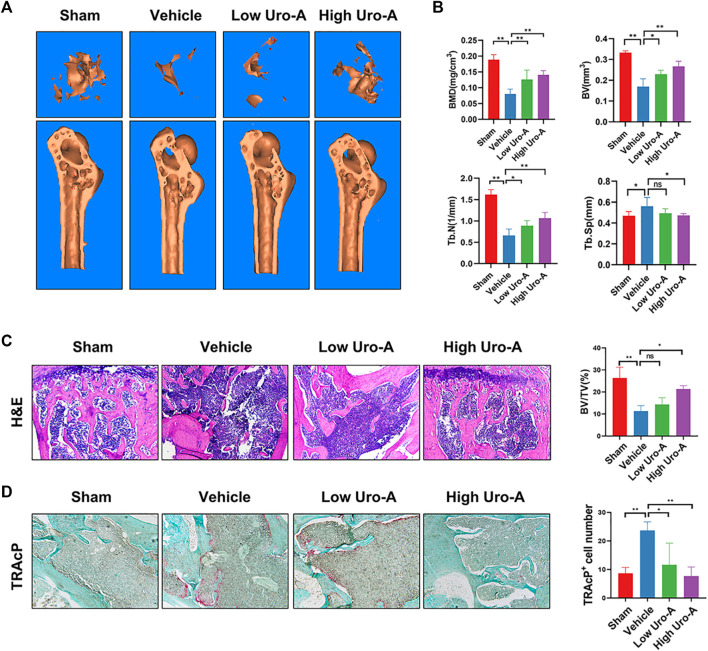
Uro-A inhibited bone loss of proximal femur in aging mice. **(A)** Micro-CT analyses of proximal femur from the indicated treatment groups. **(B)** Bone mineral density (BMD) values, bone volume (BV) values, trabecular number (Tb.N), trabecular separation (Tb.Sp) were quantified to assess bone microstructure. **(C)** H&E staining and BV/TV were conducted. **(D)** TRAcP Staining and TRAcP^+^ cell number were conducted. *n* = 5 per group. TRAcP, tartrate-resistant acid phosphatase. ns, non-significant. All bar graphs are presented as the mean ± SD. *p < 0.05, **p < 0.01 compared with the Vehicle group. ns, non-significant.

**FIGURE 7:**
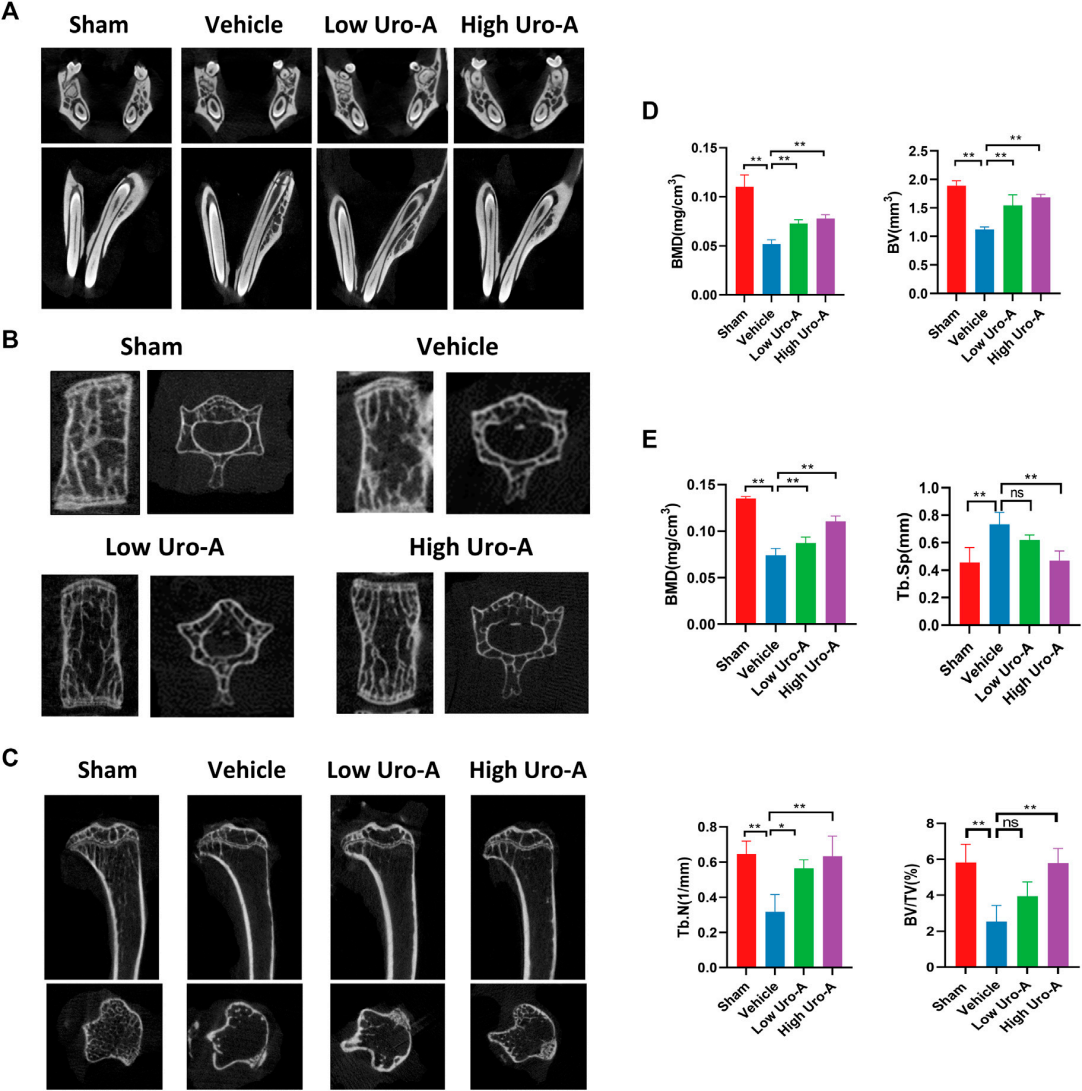
Uro-A inhibited systematic bone loss in aging mice *in vivo*. **(A)** Representative μCT images of teeth in each group, *n* = 5 per group. **(B,D)** Representative μCT images of spines in each group. Quantitative analyses of parameters regarding bone microstructure, including BMD and BV in the spine (*n* = 5 per group). **(C,E)** Representative μCT images of tibia in each group. Quantitative analyses of parameters regarding bone microstructure in tibia, including BMD, Tb.N, Tb.Sp and BV/TV (*n* = 5 per group). All bar graphs are presented as the mean ± SD. *p < 0.05, **p < 0.01 compared with the Vehicle group. ns, non-significant.

To explore the effect of Uro-A *in vivo*, C57BL/6 mice were used in our study. We purchased twenty-eight 10-week-old C57BL/6 mice and randomly divided into 4 groups. The mice fed for 20 weeks were treated as the sham group. Similarly, the mice fed to 70 weeks old were classified as the vehicle group. We administered Uro-A to mice at 50 weeks by oral administration. For the Uro-A-intervention group, mice received intragastric administration four times a week for 20 consecutive weeks. We collected the proximal femur of each group of mice. Overall, μCT analysis showed that bone mass loss was significantly higher in the vehicle group than in the sham group, and the volume of trabecular bone in the proximal femur increased after Uro-A administration relative to that in the vehicle group. Next, we tested the index of BMD, BV, Tb.N and Tb.Sp in each mouse. Bone parameter analyses revealed that BMD was significantly increased in the Uro-A administration group relative to the vehicle group (0.1259 ± 0.03 mg/cm^3^ vs. 0.08033 ± 0.02 mg/cm^3^), particularly in mice treated with a high Uro-A dose (Figures 6A,B). H&E staining confirmed that Uro-A treatment was able to maintain bone mass. Analyses of BV/TV indicated that the relative bone volume increased dramatically in the Uro-A group relative to the vehicle group (Figure 6C). Furthermore, we found that Uro-A treatment dramatically reduced the number of osteoclasts compared to that observed in vehicle group animals (Figure 6D). Quantitative analysis of TRAcP^+^ osteoclasts revealed that relative to vehicle group animals, the number of osteoclasts in the low Uro-A treatment group was reduced by almost half. In addition, we clearly observed tooth fracture at the root in the vehicle group, and Uro-A treatment greatly alleviated this condition (Figure 7A). Similarly, Uro-A dramatically reduced age-related bone loss in the tibia and spine (Figure 7B). Quantitative analysis of BMD and BV showed that both the volume and surface of bone were well maintained in the Uro-A-treated group compared with the non-Uro-A-treated sham group. Statistical analysis of bone parameters showed that high Uro-A treatment (20 mg/kg/daily) increased BMD (0.078 ± 0.005 g/cm^3^ vs. 0.052 ± 0.005 g/cm^3^) and BV (1.688 ± 0.006 mm vs. 1.124 ± 0.007 mm) in spines compared with the corresponding values in the sham group (Figure 7D). In addition, Uro-A-treated groups maintained bone mass in tibias, whereas Tb.Th remained unchanged in the current study (Figures 7C,E).

## Discussion

Aging is a physiological process characterized by body decline and the gradual loss of biological functions. The aging of the skeletal system is mainly manifested by bone loss, which is known as osteoporosis. Osteoporosis mainly includes primary osteoporosis and secondary osteoporosis. Primary osteoporosis is divided into two clinical types: postmenopausal osteoporosis and SOP. SOP often occurs in people over 70 years old and is an inevitable degenerative disease with aging. Estrogen deficiency is also one of the reasons for overall bone loss (Almeida et al., 2017). Bone loss generally involves the entire skeleton, and vertebral and hip bone mass is progressively lost with age in SOP patients (Duque et al., 2009). At present, the main measures used to treat SOP include calcium preparations and sex hormone inhibitors (Barbosa et al., 2021; Ferrari, 2021; Kim et al., 2021). Since long-term use may bring adverse reactions, it is critical to find promising and safe therapeutic schemes to treat SOP.

The homeostasis of the bone microenvironment depends on the inner interaction between osteoblasts and osteoclasts (Bellavia et al., 2021; Kondo et al., 2021; Martin et al., 2021). Histological studies have shown that SOP originates from perennial disorders of bone formation, and the excessive activation of osteoclasts participates in this process. Recent studies found that in patients suffering from SOP, the inhibitory effect on osteoclasts is weakened, but osteoclastic resorption is still active (Zhang et al., 2021). Osteoclasts contain some lysosomes, which are rich in acid proteolytic enzymes. Under pathological conditions, once the rate of bone resorption by osteoclasts exceeds the compensatory capacity of bone formation, bone loss begins and continues for a long time (Luo et al., 2021; Rauner et al., 2021). Hence, inhibiting the hyperactivity of osteoclasts is a noteworthy point for the treatment of osteoporosis.

Uro-A is recognized as a metabolic compound of intestinal flora. In recent years, it has attracted much attention because of its greatly improved biological effect on age-related degenerative diseases (Ren and Zhang, 2018; Waltz et al., 2018). Gong et al. reported that Uro-A administration decreased the levels of IL-1β, IL-6, and TNFα in an Alzheimer’s disease mouse model (Gong et al., 2019). It has also been shown to reduce apoptosis in nucleus pulposus cells and enhance autophagy in a model of intervertebral disc degeneration (Lin et al., 2020). Even in recent human clinical trials, Uro-A has been used to improve muscle strength in the elderly and improve muscle strength (Ebner et al., 2019). There are various indications that Uro-A is an effective agent for the treatment of aging-related diseases, which may even include osteoporosis. In our studies, we found that Uro-A significantly inhibited RANKL-induced osteoclastogenesis in the early stage. Osteoclast-related gene expression was significantly downregulated after Uro-A administration. We used mouse BMMs as precursor cells for inducing osteoclasts, which fully confirmed the strong anti-osteoclastic effect of Uro-A.

Autophagy is a process of maintaining cell homeostasis and is involved in the regulation of various physiological states, including the regulation of bone homeostasis. In our study, we found that Uro-A increased autophagy-related genes in BMMs. Through observation under a transmission electron microscope, we could see that some cup-shaped phagocytic vesicles appeared in the cytoplasm of BMMs. Furthermore, after stimulation with RANKL and Uro-A for 3 days, we observed that RANKL enhanced the expression of autophagy-related indicators and that Uro-A further promoted these results. Uro-A acts as an autophagy agonist, which may ameliorate the internal microenvironment of BMMs and ultimately prevent the formation of osteoclasts.

Classically, the MAPK signaling pathway is highly involved in osteoclast differentiation and actively regulates osteoclast maturity (Fang et al., 2021; Yang et al., 2021). To explore the response mechanism by which Uro-A inhibits osteoclasts, we tested the expression of the MAPK family after Uro-A treatment. The results showed that the activation of phosphorylated protein was reversed with Uro-A treatment in a concentration-dependent manner. Uro-A has been verified to inhibit MAPK activation in LPS stimulated BMMs. Furthermore, Ding et al. has introduced that Uro-A suppressed IL-1β-induced inflammation responses and cartilage degradation via MAPK signaling pathway. The MAPK family is responsible for the translocation of NFATc1 to drive osteoclast lineage, which may explain why Uro-A could effectively inhibit osteoclast information *in vitro*. In previous research, Li et al. (2017) found that curcumin suppressed chondrocyte apoptosis by enhancing autophagy *via* ERK cascades and that ERK signaling is a key upstream regulator of Beclin 1. Similarly, ERK/P38 has been confirmed to play a critical role in Beclin 1-mediated inhibition of RANKL-induced osteoclastogenesis (Tong et al., 2021). In view of this, future research regarding the role of MAPK signaling in autophagy is thus warranted.

Our previous study showed that Uro-A could alleviate bone loss in the distal femur of ovariectomized mice (Tao et al., 2021), whether Uro-A could be used to treat age-induced systemic bone loss is of great significance. Statistical analysis of bone parameters in an *in vivo* animal model revealed that Uro-A increased BMD and BV in the sections of the proximal femur, spine and tibia compared with the non-Uro-A-treated group. *In vivo*, Uro-A effectively inhibited osteoclast activation in the proximal femur. However, bone biology is a complex process that demands crosstalk among many cells. Future research regarding the effect of Uro-A in osteoblasts needs to be explored. Even so, it is worth noting that bone loss in senile osteoporosis mainly occurs in the vertebral body and hip. Conceivably, Uro-A nutritional supplementation may be a promising therapeutic scheme for senile osteoporosis by inhibiting osteoclast formation.

## Conclusion

In summary, our work highlighted the therapeutic effect of Uro-A on senile osteoporosis. We found that Uro-A inhibited RANKL-induced osteoclastogenesis by suppressing MAPK cascades *in vitro*. Additionally, Uro-A administration enhanced the autophagy capability of BMMs and maintained cellular homeostasis. Furthermore, Uro-A eased bone loss in multiple parts of aging mice. Bone biology is a complex process that demands coordinated activities between osteoblastic formation and osteoclastic resorption, and the effect of Uro-A on osteoblasts should be investigated in the future. Conceivably, Uro-A could be a qualified and promising regulator for maintaining bone mass via its powerful anti-osteoclast activity.

## Data Availability

The original contributions presented in the study are included in the article/Supplementary Material, further inquiries can be directed to the corresponding authors.

## Abbreviations

SOP, senile osteoporosis; Uro-A, urolithin a; Micro-CT, micro-computed tomography; RANKL, receptor activator for nuclear factor-κB ligand; BMD, bone mineral density; BV, bone valume; BV/TV, bone volume per tissue volume; Tb N, Trabecular number; Tb Sp, trabecular separation; Ct Th, cortical thickness; PCR, polymerase chain reaction; PBS, phosphate buffered saline; MAPK, mitogen-activated protein kinase; ET, ellagitannins; EA, ellagic acid; LPS, Lipopolysaccharide; M-CSF, macrophage colony stimulating factor; TRAcP, tartrate-resistant acid phosphatase; H&E, hematoxylin and eosin; CTSK, Cathepsin K; NFATc1, nuclear factor of activated T cells 1; Acp5, acid phosphatase 5; MMP 9, matrix metallopeptidase 9; TEM, transmission electron microscopy.
